# Selecting a Subset Based on the Patient-Reported Outcomes Version of the Common Terminology Criteria for Adverse Events for Patient-Reported Symptom Monitoring in Lung Cancer Treatment: Mixed Methods Study

**DOI:** 10.2196/26574

**Published:** 2021-09-14

**Authors:** Evalien Veldhuijzen, Iris Walraven, José Belderbos

**Affiliations:** 1 Department of Radiation Oncology Netherlands Cancer Institute Amsterdam Netherlands; 2 Department for Health Evidence Radboud University Medical Center Nijmegen Netherlands

**Keywords:** PRO-CTCAE, lung cancer, side effects, patient-reported outcomes, PROM, symptomatic adverse events

## Abstract

**Background:**

The Patient-Reported Outcomes Version of the Common Terminology Criteria for Adverse Events (PRO-CTCAE) item library covers a wide range of symptoms relevant to oncology care. There is a need to select a subset of items relevant to specific patient populations to enable the implementation of PRO-CTCAE–based symptom monitoring in clinical practice.

**Objective:**

The aim of this study is to develop a PRO-CTCAE–based subset relevant to patients with lung cancer that can be used for monitoring during multidisciplinary clinical practice.

**Methods:**

The PRO-CTCAE–based subset for patients with lung cancer was generated using a mixed methods approach based on the European Organization for Research and Treatment of Cancer guidelines for developing questionnaires, comprising a literature review and semistructured interviews with both patients with lung cancer and health care practitioners (HCPs). Both patients and HCPs were queried on the relevance and impact of all PRO-CTCAE items. The results were summarized, and after a final round of expert review, a selection of clinically relevant items for patients with lung cancer was made.

**Results:**

A heterogeneous group of patients with lung cancer (n=25) from different treatment modalities and HCPs (n=22) participated in the study. A final list of eight relevant PRO-CTCAE items was created: decreased appetite, cough, shortness of breath, fatigue, constipation, nausea, sadness, and pain (general).

**Conclusions:**

On the basis of the literature and both professional and patient input, a subset of PRO-CTCAE items has been identified for use in patients with lung cancer in clinical practice. Future work is needed to confirm the validity and effectiveness of this PRO-CTCAE–based lung cancer subset internationally and in real-world clinical practice settings.

## Introduction

### Background

Lung cancer is the most common cancer in men and the third most common cancer in women worldwide [1]. Treatment options are often multidisciplinary, including surgery, chemotherapy, radiation therapy, targeted therapy, immunotherapy, or a combination of these treatments [2]. Owing to both the tumor and the (combination of) treatments, patients can experience a wide range of symptoms and toxicities that impair their health-related quality of life (HRQoL) and require careful management [3]. Historically, toxicities have been rated by health care practitioners (HCPs) most typically using the Common Terminology Criteria for Adverse Events (CTCAE), which is broadly implemented to monitor toxicity in oncology trials and clinical care [4]. The concept of *clinician scoring* has recently been challenged by a number of studies that have observed relatively high levels of disagreement between toxicities reported by clinicians and patients [5-7].

Patient-reported outcome measures (PROMs) have been demonstrated to improve patient-clinician communication about symptoms and are therefore increasingly recognized as an important source of information in clinical decision-making [8-10]. PROMs could also function as a tool for routine toxicity management as part of clinical care. In two previous randomized trials, Basch et al [11] and Denis et al [12] used a selection of patient-reported symptoms to monitor symptoms during chemotherapy in patients with metastatic cancer and lung cancer, respectively. These trials have shown that PROM symptom monitoring not only improves symptom management but also significantly improves HRQoL and overall survival [11-14]. Potential underlying mechanisms for these positive results include an earlier and therefore more effective response to progressively evolving symptoms, including timely initiation of supportive treatments, dose modifications, and early referrals [11,12,15,16].

On the basis of the results of these trials, there has been a growing call for the development and implementation of standardized patient-reported symptom monitoring tools for use in both clinical research and clinical practice [14,17]. A major advance in this direction has been the development and testing of the US National Cancer Institute’s Patient-Reported Outcome Version of the CTCAE (PRO-CTCAE). The PRO-CTCAE is developed through a consortium of patient-reported outcome (PRO) researchers, clinical investigators, trial sponsors, patient advocates, and the Food and Drug Administration (FDA), and it comprises 124 items, based on 78 CTCAE toxicities considered appropriate for patient reporting [18]. These items have been comprehensively validated in English-speaking patients [19] and have been translated and linguistically validated in a large number of languages, including Dutch [20].

Frequent administration of the complete library of PRO-CTCAE items is considered impractical and burdensome [18,21]. However, this validated item library of symptoms can form the basis of a PRO monitoring subset. Conforming to what the FDA has described, the selection of a relevant item set is of critical importance to provide insights into the most important toxicities for the treatments that are being evaluated [22]. Several studies have focused on creating a subset of PRO-CTCAE. Examples include an item subset for patients with bladder cancer receiving chemotherapy and immunotherapy, and a subset of patients receiving immunotherapy in metastatic melanoma [23-25]. Similar to most cancer diagnoses, lung cancer is often treated in a multidisciplinary setting, including a treatment plan for multiple modalities and a variety of involved health care professionals [26]. These multidisciplinary teams can use PROMs to improve the collective monitoring of patients [27].

### Objective

The aim of this study is to systematically develop a multidisciplinary subset of PRO-CTCAE items that are clinically relevant for patients with lung cancer and that can be used for monitoring during multidisciplinary clinical practice.

## Methods

### Item Identification

**Figure 1:**
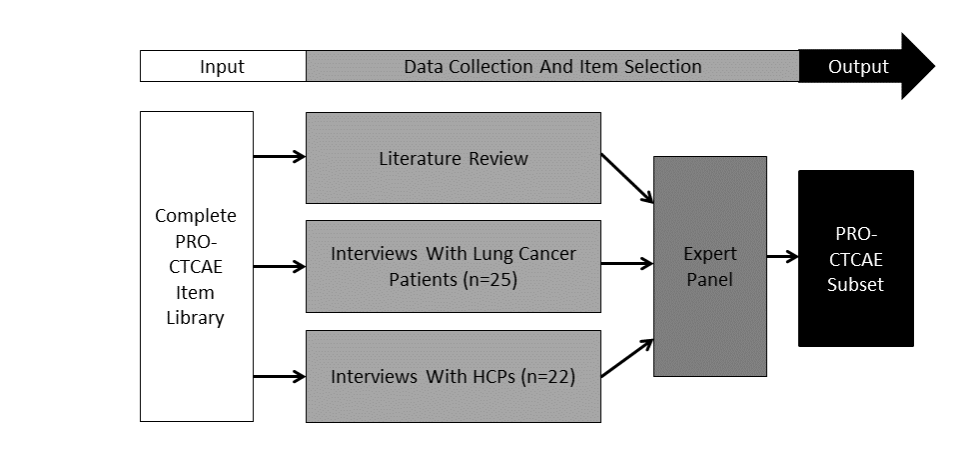
A schematic overview of the item identification process. HCP: health care practitioner; PRO-CTCAE: Patient-Reported Outcomes Version of the Common Terminology Criteria for Adverse Events.

A schematic overview of the subset identification method is presented in Figure 1. The original PRO-CTCAE item library was the main source and starting point for the development of the lung cancer subset. The procedure to identify relevant items for the subset approximates phase 1 of the European Organization for Research and Treatment of Cancer (EORTC) Quality of Life Group guidelines for developing PROMs [28]. Three sources were used to compile the relevant item list.

First, a literature search was conducted to ensure the identification of all relevant toxicities from both the literature and existing questionnaires. We used the PubMed database, with the following search terms: *non-small cell lung cancer* *OR* *small cell lung cancer AND adverse events OR toxicities OR symptoms OR side effects AND Chemotherapy OR Radiation OR Chemo radiation OR Immunotherapy OR targeted therapy OR surgery*. From these results, the literature that included existing questionnaires and reviews on lung cancer toxicity was selected. Next, we identified the PRO-CTCAE items that corresponded to the symptoms derived from the literature. The literature study was conducted as comparative evidence to be used complementary to the data collected from the patients and HCPs.

Second, the patients’ perspectives were included to ensure content validity [29]. A heterogeneous sample (n=25) of patients with lung cancer was invited to participate in a semistructured interview. Patients were recruited from the Netherlands Cancer Institute. We used purposive sampling to include patients varying in terms of age, sex, stage, and treatment type. The eligibility criteria were as follows: aged ≥18 years; able to provide informed consent; either currently undergoing lung cancer treatment (at least 2 weeks after the start of treatment) or in follow-up (having completed lung cancer treatment within the previous 6 months); and basic fluency in the Dutch language. The exclusion criteria were psychological or cognitive problems as judged by the HCP, which would interfere with participating in an interview.

Finally, the HCPs working in the Netherlands Cancer Institute (n=22) in the field of lung cancer, including pulmonologists (n=4), radiation oncologists (n=12), thoracic surgeons (n=3), and nurse specialists or physician assistants (n=3) specialized in the treatment of lung cancer, were invited for an interview to provide their opinions about the most relevant items to be included in a lung cancer subset.

### Interview Procedures

A scripted interview guide was used based on the EORTC guidelines [28]. During the first part of the interview, patients were asked to freely describe their experiences and symptoms. Subsequently, patients were asked to complete the PRO-CTCAE item library by filling out a questionnaire that included all items. This was followed by a debriefing interview to determine what the experienced symptoms meant to the patient, the extent to which patients had experienced the symptoms, and if they had experienced any symptoms not included in the questionnaire. Patients were encouraged to comment on the PRO-CTCAE symptom terms and were asked to rate each symptom for relevance using a scale of 1 (not relevant) to 4 (very relevant) [28]. Patients were asked to select a maximum of 10 most impactful symptoms to assess the importance of the PRO-CTCAE symptoms. Finally, patients were asked to identify symptoms that should definitely be included or excluded.

The HCPs took part in a semistructured interview in which they were shown the complete PRO-CTCAE item list and were asked if (1) there were symptoms included that the medical specialists considered clinically relevant (scored as 1) or irrelevant (scored as 2; in terms of treatability and urgency) for patients with lung cancer and (2) if symptoms were missing from the list that they considered relevant. The reasons for relevance or irrelevance were specified.

### Item Selection

An overview table was created based on the complete PRO-CTCAE item library, in which the results of the data collection were collected and ranked. For literature data, the prevalence of the item in the included literature sources was calculated (*literature score).* Next, for patient data, the mean relevance score for each PRO-CTCAE item was calculated *(patient relevance score)*. The top 10 items (based on the *patient relevance score*) were reviewed and compared to gain insight into the different treatment modalities. Finally, for the HCP data, the percentage of HCPs who rated it as relevant was calculated *(HCP relevance score)*. The table was then sorted from high to low using the *patient relevance score* data as the primary rank, followed by the *HCP relevance score* and the *literature score.*

For the final item selection, the ranked list of items was reviewed by an expert review panel, including a pulmonologist, a radiation oncologist, an epidemiologist, and two public health experts. During this process, the relevance scores of the patients were of primary importance in the selection of items. The expert review consisted of three rounds. First, all items with a low patient relevance score (<2) were reviewed. Next, items with a high patient relevance score (>2.5) were reviewed. The third round consisted of a review of items with a relevance score between 2 and 2.5. Decisions to include or exclude items from the final list were based on the following features: (1) lack of clinical relevance (in terms of treatability and urgency), (2) upsetting items, and (3) redundancy (multiple closely related items) [28]. As the goal was to generate a subset of items most relevant for clinical practice without creating excessive respondent burden, the item that was indicated most relevant by the patients was chosen in case of redundancy (eg, fatigue and insomnia). The discussions continued until a consensus was reached over the final item selection.

## Results

### The Literature

Table 1 provides an overview of the selected studies. Relevant literature included the following existing questionnaires: the EORTC Quality of Life core questionnaire (QLQ) C30 and the EORTC QLQ Lung Cancer module (EORTC QLQ LC13), the Development of the Functional Assessment of Cancer Therapy-Lung, and the MD Anderson Symptom Inventory for lung cancer [30-33]. More recent efforts to define relevant patient outcomes in lung cancer by Mak et al [34], Reeve et al [35], and Koller et al [36] were included as well. From the study by Koller et al [36], we included a list of quality of life issues as rated by patients and HCPs in phase 1 of the EORTC Module Development Guidelines [28].

**Table 1:** The Patient-Reported Outcomes Version of the Common Terminology Criteria for Adverse Events items that were identified in the literature for each included source.

PRO-CTCAE^a^ item	Study					
	Aaronson et al^b^ [30]	Cella et al^c^ [32]	Cleeland et al^d^ [33]	Koller et al^e^ [36]	Mak et al^f^ [34]	Reeve et al^g^ [35]
Fatigue	✓^h^	✓	✓	✓	✓	✓
Shortness of breath	✓	✓	✓	✓	✓	✓
Cough					✓	
Decreased appetite	✓	✓	✓			
Pain	✓	✓		✓	✓	✓
Dizziness	✓			✓		
Constipation	✓		✓			✓
Insomnia	✓	✓	✓			✓
Nausea	✓	✓	✓	✓		
Rash				✓		
Sadness	✓	✓	✓			✓
Difficulty swallowing	✓			✓		
Decreased sexual interest				✓		
Diarrhea						✓
Anxious	✓					
Hoarseness	✓					
Vomiting	✓		✓			
Numbness and tingling	✓		✓	✓		
Memory			✓	✓		✓
Concentration	✓					✓
Voice quality changes	✓					
Hair loss	✓	✓				
Acne				✓		
Nail loss				✓		
Nail ridging				✓		
Nail discoloration				✓		

^a^PRO-CTCAE: Patient-Reported Outcomes Version of the Common Terminology Criteria for Adverse Events.

^b^Development of the European Organization for Research and Treatment of Cancer (EORTC) Quality of Life core questionnaire (QLQ) C30 and the EORTC QLQ Lung Cancer module (EORTC QLQ LC13).

^c^Development of the Functional Assessment of Cancer Therapy-Lung.

^d^Development of MD Anderson Symptom Inventory for lung cancer.

^e^Based on the phase 1 study of the international study to revise the European Organization for Research and Treatment of Cancer questionnaire for assessing quality of life in lung cancer patients.

^f^Delphi study with health care professionals in the field of lung cancer.

^g^Systematic literature review and expert panel.

^h^Item present.

The following 24 PRO-CTCAE items were identified from the selected literature: *fatigue, shortness of breath, decreased appetite, pain, dizziness, constipation, insomnia, nausea, sad or discouraged, difficulty swallowing, anxious, hoarseness, vomiting, numbness and tingling, concentration, voice quality changes, hair loss, memory, rash, decreased libido, acne, nail loss, nail ridging,* and *nail discoloration*.

### Patient Interviews

Table 2 presents an overview of the patients and treatment characteristics. The mean age of the patients was 66 years (SD 8). The stage distribution was as follows: stage I, 8% (2/25); stage II, 12% (3/25); stage III, 36% (9/25); and stage IV, 44% (11/25). A broad range of treatment modalities (radiotherapy, 3/25, 12%; stereotactic radiotherapy, 2/25, 8%; concurrent chemotherapy and radiation, 5/25, 20%; surgery, 5/25, 20%; and systemic treatment such as chemotherapy, 1/25, 4%; immunotherapy, 6/25, 24%; and targeted therapy, 3/25, 12%) were included.

**Table 2:** Characteristics of all patients participating in the item selection interviews (n=25).

Patient characteristics					Values
**Gender, n (%)**					
	Female			13 (48)	
	Male			12 (52)	
Age (years), mean (SD; range)					66 (8; 55-79)
**Lung cancer stage, n (%)**					
	Stage I			2 (8)	
	Stage II			3 (12)	
	Stage III			9 (36)	
	Stage IV			11 (44)	
**Treatment modality, n (%)**					
	**Surgery**			5 (20)	
		Radiotherapy	3 (12)		
		Stereotactic radiotherapy	2 (8)		
	Concurrent chemoradiation			5 (20)	
	**Systemic treatments**				
		Chemotherapy	1 (4)		
		Immunotherapy	6 (24)		
		Targeted therapy	3 (12)		
**Treatment status, n (%)**					
	On treatment			14 (56)	
	<1 month posttreatment			9 (36)	
	1-3 months posttreatment			2 (8)	
**Highest level of education, n (%)**					
	Primary school			3 (12)	
	Lower vocational education			2 (8)	
	High school			9 (36)	
	Higher vocational education			6 (24)	
	Scientific education			5 (20)	

Fatigue was scored as the most relevant symptom from the patient’s perspective, with a *patient relevance score* of 85.7. Seven other items were scored above 2.5 (*shortness of breath, cough, insomnia, decreased appetite, dizziness, constipation, nausea, and sadness*).

When reviewing the items per treatment modality, the top 10 items per modality category (radiotherapy, systemic treatment, concurrent chemoradiation, and surgery) were compared. *Fatigue*, *shortness of breath*, and *cough* overlapped in all modalities, and *dizziness*, *hives*, and *constipation* overlapped in three out of four modalities. Some items were present in two of the four categories, including *sadness* for systemic therapy and surgery, *itchy skin* and *joint pain* for both radiotherapy and surgery, and *insomnia* in concurrent radiation and surgery. Although most items overlapped between categories, the different treatment modalities seemed to influence the type of symptoms that were described as most relevant by the patients. Radiotherapy-specific symptoms included *taste changes*, *dry skin*, *headache*, and *bruises*. The concurrent chemoradiation-specific symptoms were *urinary frequency*, *heart palpitations*, and *difficulty swallowing*. Items specifically relevant for systemic treatment (ie, chemotherapy, immunotherapy, and targeted therapy) were *discouraged*, *anxiety*, and *nausea*. Finally, patients treated with surgery described *flatulence* and *achieve and maintain an erection*.

### HCP Interviews

The participating HCPs reported having experience with a variety of treatment modalities. They had experience with chemoradiation 77% (17/22), immunotherapy 23% (5/22), surgery 18% (4/22), radiotherapy 59% (13/22), chemotherapy 18% (4/22), and experimental or targeted therapies 9% (2/22). Of the participating HCPs, 36 items had an HCP relevance score <50%. The items that were identified as most relevant by the HCPs included *shortness of breath, wheezing, fatigue, decreased appetite, nausea, difficulty with swallowing, vomiting,* and *headache*.

### Item Selection

**Figure 2:**
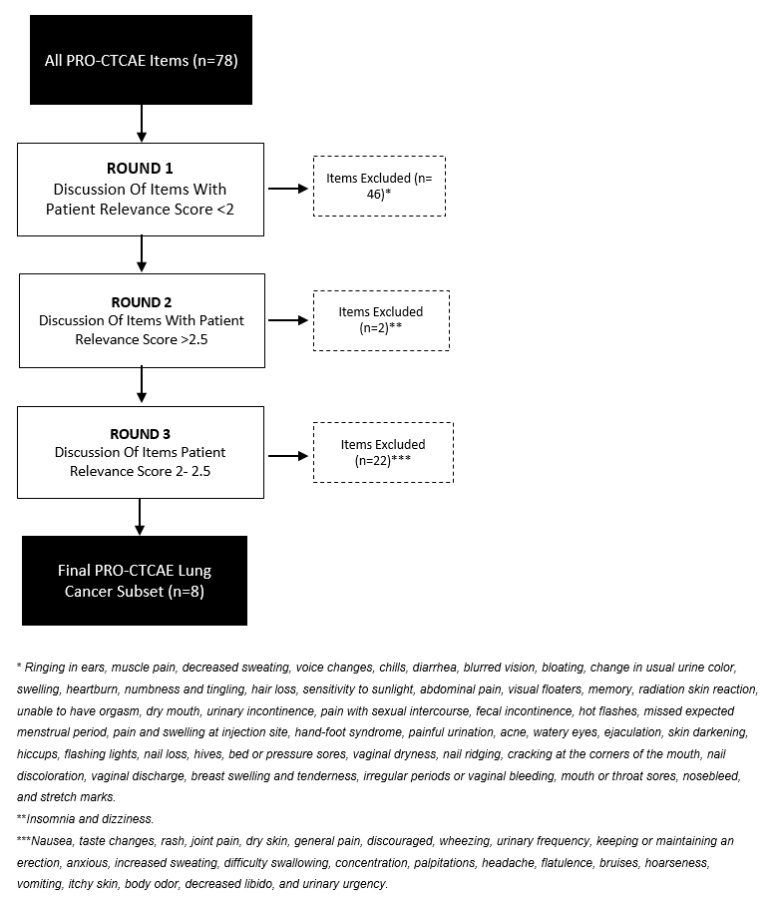
Flowchart of the item selection process. PRO-CTCAE: Patient-Reported Outcomes Version of the Common Terminology Criteria for Adverse Events.

All PRO-CTCAE items were ranked by *patient relevance score* first followed by *HCP relevance score* and finally *literature score*, which is shown in Multimedia Appendix 1*.* An overview of the item selection process is shown in the flowchart in Figure 2. In round 1, 46 items were excluded. These items had a low *patient relevance score* of <2. The expert panel collectively agreed to eliminate these items. In round 2, items with a high patient relevance score of >2.5 were discussed for inclusion. This list consisted of eight items, including *fatigue*, *shortness of breath*, *cough*, *insomnia*, *decreased appetite*, *dizziness*, *constipation*, and *sadness*. Experts agreed to include all these items with the exception of *insomnia* and *dizziness*. The item *fatigue* was chosen over *insomnia* because fatigue covers more than insomnia, and these items are known to be highly correlated. Furthermore, *fatigue* was scored more frequently across all data sources than *insomnia* (patients, HCPs, and the literature). There was no expert consensus on the inclusion of *dizziness* based on clinical relevance. Moreover, this item scored relatively low on the *HCP relevance score* and the *literature score* compared with the other items in round 2 and was therefore not included.

In round 3, 23 items with a *patient relevance score* between 2 and 2.5 were discussed. Uniform agreement for exclusion of the following items was reached: *urinary urgency*, *decreased libido, body odor*, *itchy skin*, *flatulence*, *concentration*, *increased sweating*, *achievement and maintenance of erection*, *urinary frequency*, and *dry skin*. The remaining items were discussed by the expert panel. Of these items, *nausea* was ranked the highest with a *patient relevance score* of 2.42, an *HCP relevance score* of 71.4, and a *literature score* of 50. On the basis of these scores and the judgment of clinical relevance, the expert panel decided to include this item in the final list. The item *taste changes* and *difficulty swallowing* were excluded because of their correlation with the higher-ranked item *decreased appetite*. The item *rash* was excluded because of the low level of clinical relevance based on the expert panel, as well as the low *literature score* (12.5). The item *joint pain* had a *higher patient relevance* score than the item for *general pain* (2.33 and 2.29, respectively). However, in light of the clinical use of the questionnaire, the item *general pain* was preferred because it would cover more than solely joint pain and was therefore included in the list. The items *discouraged* and *anxious* were excluded because the higher ranking and the correlating item *sadness* was already included. The item *wheezing* was excluded because the higher ranking and correlation item *shortness of breath* was already included in round 2.

Therefore, the final list of eight items included *fatigue, cough, shortness of breath, decreased appetite, constipation, nausea, general pain,* and *sadness*.

## Discussion

### Principal Findings

To our knowledge, this is the first study to develop a PRO-CTCAE–based subset for the PRO-based monitoring of toxicity in patients with lung cancer. This subset enables the incorporation of patient perspectives in clinical monitoring of patients with lung cancer using the well-established and FDA-endorsed PRO-CTCAE item library. This study may serve as an example for the future development of other site-specific PRO-CTCAE–based subsets for symptom monitoring in clinical practice.

Previously, there have been successful efforts of PRO-based monitoring in several trials showing improved symptom management, HRQoL, and even overall survival [11-14]. Basch et al [11] performed a trial (n=766) testing PRO-based monitoring in patients treated with chemotherapy for advanced solid tumors. This trial used a list of 12 common symptoms based on previous literature [37]. Denis et al [38] performed a similar PRO-based monitoring trial in patients with lung cancer (n=121). Both author groups did not report on the development of the symptoms list, but they performed a study on the capability of symptom lists to detect lung cancer relapse [38]. When comparing our final item list with the lists used by Denis et al [38] and Basch et al [11], our study results seem to be fairly consistent with the symptom lists used in these successful PRO-based monitoring trials. In total, 50% (4/8) of items are listed in both lists, that is, cough, shortness of breath, decreased appetite, pain, and fatigue (Denis et al [38] used weakness) [14,17]. Two items, constipation and nausea, were only listed by Basch et al [17]. Finally, sadness was not a part of the two PRO-based monitoring trials. Sadness and depression are however closely related and often sadness may indicate an occurrence or development of depression [14,17]. Items that were not included in our list because they were not included in the PRO-CTCAE item library were *fever, facial swelling, lump under the skin, voice changes coughing up blood,* and *body weight.* Other items that did not correspond with our subset were *painful urination, diarrhea, hot flashes,* and *tingling*, which were included in the more heterogeneous trial of Basch et al [17].

The study results are also in line with previous efforts of creating a symptom subset. A recent study created anatomic site-specific PRO-CTCAE item sets, including items for thorax radiation [39]. Their results were based on 30 patients who received thoracic radiation (including 16 patients with lung cancer). Their proposed relevant item set is in line with the results of this study [39]. Few previous studies have systematically selected a subset of PRO-CTCAE items [23,25]. Nissen et al [25] specifically aimed at three types of drugs and their adverse events in the treatment of metastatic prostate cancer, which were mainly based on FDA, European Medicine Agency, and randomized controlled trial reports and included relatively small samples of patients’ interviews (n=16). This resulted in a relatively large subset of 25 PRO-CTCAE items compared with this study. Even though the number of items tested by Nissen et al [25] was considered feasible, they were tested in a setting of a one-time measurement only. Therefore, the presented subset might be a more feasible choice for weekly monitoring over a longer period. Moreover, the length of the subset was also comparable with the questionnaires used in previous successful trials that performed weekly monitoring [11,14]. Furthermore, this study, along with other studies, was performed in different target populations with slightly different aims, for example, Tolstrup et al [24] focused on immunotherapy in metastatic melanoma, and the study by Taarnhøj et al [23] focused on chemo- and immunotherapy for bladder cancer, whereas in this study, it was a requirement from our sponsor of Dutch medical specialists involved in the treatment of patients with lung cancer to have the same questionnaire for the (often) multimodality lung cancer treatment.

### Strengths and Limitations

The key strength of this study is the use of a mixed method approach that includes the literature, patients, and expert perspectives. This study included a patient sample that reflected the stage distribution of the lung cancer population and included a variety of treatment modalities that are frequently used in clinical practice [40]. This multidisciplinary subset allows the monitoring of patient symptoms during the entire treatment course and therefore facilitates implementation within clinical care settings [41]. The emphasis on the patient and expert perspective may facilitate the implementation of PROMs within clinical cancer care, so the chance that this subset is perceived as valuable to the clinician is more likely to enable successful clinical implementation [16].

Our subset is a valuable PRO tool because it enables reliable remote monitoring, which can help provide necessary care to patients while minimizing the use of health care facilities. Remote monitoring using directly integrated PROM results in the electronic health record is expected to be successful because it minimizes barriers for use within the daily clinical routine [42].

The findings of this study are subject to several limitations. A methodological choice to let patients rate the items on a scale of 1 to 4, as opposed to the binary HCP rating that was used in this study, might have influenced the comparability of both results. However, we believe the 1 to 4 scale gives the patients a tool to rate the relative importance of their own experience, where an HCP can judge relevance or irrelevance based on a large number of cases and expertise.

For this subset, a set of items that were intended as a core set for multidisciplinary use were selected. Despite the advantages of multidisciplinary use and implementation, one could argue that the treatment experience may vary based on the different treatment modalities, and this could cause symptoms to be missed in patients depending on the specific treatment that is given. The results of this study showed that some symptoms were experienced as specifically relevant for distinct treatment modalities. One may consider adding these treatment-specific items when the subset is solely used in the context of one treatment modality. Moreover, we encouraged the use of the PRO-CTCAE’s *other symptoms* item, in which a patient can freely report and score additional symptoms.

An arguable weakness that needs to be considered when interpreting these findings is that this study was designed as an EORTC phase 1 or 2 study to develop guidelines for developing PROMs (hence the relatively small sample). This entails that in order to make statements with regard to psychometric properties, acceptability, and compliance, further international field testing is needed. This study was a single-center study, and multicenter verification is certainly needed. Nonetheless, the PRO-CTCAE item library has proven to be a valid and reliable questionnaire in previous studies, and it has only recently been linguistically validated in Dutch-speaking patients [19,20]. Moreover, the PRO-CTCAE subset with the additional items of body weight and temperature and a specification of *coughing up blood* is currently being tested in the trial *SYMPRO-Lung* (Symptom Monitoring With Patient-Reported Outcomes Using a Web Application Among Lung Cancer Patients in the Netherlands; Netherlands Trial Register: NL7897).

### Conclusions

This study presents a subset of PRO-CTCAE items for multidisciplinary PRO monitoring of patients with lung cancer during and after treatment. The results of the final item selection were considered relevant for monitoring patients with lung cancer. Continued efforts are needed to further validate the psychometric properties and the value of the PRO-CTCAE lung cancer subset in real-world clinical practice.

## Appendix

Multimedia Appendix 1 Relevance scores for all Patient-Reported Outcomes Version of the Common Terminology Criteria of Adverse Events items in order of relevance.

## Abbreviations

CTCAE: Common Terminology Criteria for Adverse Events
EORTC: European Organization for Research and Treatment of Cancer
FDA: Food and Drug Administration
HCP: health care practitioner
HRQoL: health-related quality of life
PRO: patient-reported outcome
PRO-CTCAE: Patient-Reported Outcomes Version of the Common Terminology Criteria for Adverse Events
PROM: patient-reported outcome measure
QLQ: Quality of Life core questionnaire
SYMPRO-Lung: Symptom Monitoring With Patient-Reported Outcomes Using a Web Application Among Lung Cancer Patients in the Netherlands
