# Supplementary material for: Selecting a Subset Based on the Patient-Reported Outcomes Version of the Common Terminology Criteria for Adverse Events for Patient-Reported Symptom Monitoring in Lung Cancer Treatment: Mixed Methods Study
Source: JMIR Cancer. 2021 Sep 14;7(3):e26574. doi: 10.2196/26574 (PMC8479599; doi:10.2196/26574)
Supplement: Multimedia Appendix 1 [file cancer_v7i3e26574_app1.docx]

**Table S1**. Relevance scores for all PRO-CTCAE items in order of relevance.

|  | PRO-CTCAE items | Patient relevance score | HCP relevance score | Literature relevance score |
| --- | --- | --- | --- | --- |
| Round 2 | **Fatigue*** | 3.13 | 85.7 | 87.5 |
|  | **Shortness of breath*** | 2.8 | 100 | 100 |
|  | **Cough*** | 2.7 | 95.2 | 87.5 |
|  | Sleeplessness | 2.7 | 66.7 | 37.5 |
|  | **Decreased appetite*** | 2.6 | 85.7 | 75.0 |
|  | Dizziness | 2.6 | 42.9 | 25.0 |
|  | **Constipation*** | 2.5 | 52.4 | 50.0 |
|  | **Sadness*** | 2.5 | 42.9 | 50.0 |
| Round 3 | **Nausea*** | 2.4 | 71.4 | 50.0 |
|  | Taste changes | 2.4 | 55.6 | 0 |
|  | Rash | 2.4 | 70.0 | 12.5 |
|  | Joint pain | 2.3 | 44.4 | 0 |
|  | Dry skin | 2.3 | 38.1 | 0 |
|  | **General pain*** | 2.3 | 66.7 | 75.0 |
|  | **Discouraged*** | 2.3 | 42.9 | 37.5 |
|  | Wheezing | 2.3 | 77.8 | 0 |
|  | Urinary frequency | 2.3 | 33.3 | 0 |
|  | Keeping or maintaining an erection | 2.2 | 22.2 | 12.5 |
|  | Anxious | 2.2 | 57.1 | 12.5 |
|  | Increased sweating | 2.2 | 33.3 | 0 |
|  | Difficulty swallowing | 2.2 | 81.0 | 37.5 |
|  | Concentration | 2.2 | 47.6 | 25.0 |
|  | Palpitations | 2.1 | 55.6 | 0 |
|  | Headache | 2.1 | 70.0 | 0 |
|  | Flatulence | 2.1 | 44.4 | 0 |
|  | Bruises | 2.0 | 55.6 | 0 |
|  | Hoarseness | 2.0 | 77.8 | 12.5 |
|  | Vomiting | 2.0 | 76.2 | 37.5 |
|  | Itchy skin | 2.0 | 44.4 | 0 |
|  | Body odor | 2.0 | 44.4 | 0 |
|  | Decreased libido | 2.0 | 28.6 | 12.5 |
|  | Urinary urgency | 2.0 | 22.2 | 0 |
| Round 1 | Ringing in ears | 1.9 | 55.6 | 0 |
|  | Muscle pain | 1.9 | 44.4 | 12.5 |
|  | Decreased sweating | 1.9 | 33.3 | 0 |
|  | Voice changes | 1.9 | 66.7 | 25 |
|  | Chills | 1.9 | 55.6 | 0 |
|  | Diarrhea | 1.9 | 33.3 | 12.5 |
|  | Blurred vision | 1.8 | 55.6 | 0 |
|  | Bloating | 1.8 | 55.6 | 0 |
|  | Change in usual urine color | 1.8 | 22.2 | 0 |
|  | Swelling | 1.9 | 66.7 | 0 |
|  | Heartburn | 1.8 | 57.1 | 0 |
|  | Numbness and tingling | 1.8 | 47.6 | 62.5 |
|  | Hair loss | 1.8 | 42.9 | 12.5 |
|  | Sensitivity to sunlight | 1.8 |  | 0 |
|  | Abdominal pain | 1.8 | 55.6 | 0 |
|  | Visual floaters | 1.8 | 44.4 | 0 |
|  | Memory | 1.8 | 55 | 50 |
|  | Radiation skin reaction | 1.8 | 66.7 | 0 |
|  | Unable to have orgasm | 1.8 | 22.2 | 12.5 |
|  | Dry mouth | 1.8 | 33.3 | ? |
|  | Urinary incontinence | 1.7 | 22.2 | 0 |
|  | Pain with sexual intercourse | 1.7 | 33.3 | 12.5 |
|  | Fecal incontinence | 1.7 | 22.2 | 12.5 |
|  | Hot flashes | 1.7 | 55.6 | 0 |
|  | Missed expected menstrual period | 1.7 | 22.2 | 0 |
|  | Pain and swelling at injection site | 1.7 | 55.6 | 0 |
|  | Hand-foot syndrome | 1.7 | 44.4 | 0 |
|  | Painful urination | 1.6 | 33.3 | 0 |
|  | Acne | 1.6 | 28.6 | 12.5 |
|  | Watery eyes | 1.6 | 33.3 | 0 |
|  | Ejaculation | 1.6 | 22.2 | 12.5 |
|  | Skin darkening | 1.5 | 55.6 | 0 |
|  | Hiccups | 1.5 | 44.4 | 0 |
|  | Flashing lights | 1.5 | 44.4 | 0 |
|  | Nail loss | 1.5 | 33.3 | 12.5 |
|  | Hives | 1.5 | 44.4 | 0 |
|  | Bed/pressure sores | 1.4 | 44.4 | 0 |
|  | Vaginal dryness | 1.4 | 22.2 | 0 |
|  | Nail ridging | 1.4 | 9.50 | 12.5 |
|  | Cracking at the corners of the mouth | 1.4 | 42.9 | 0 |
|  | Nail discoloration | 1.4 | 14.3 | 12.5 |
|  | Vaginal discharge | 1.4 | 22.2 | 0 |
|  | Breast swelling and tenderness | 1.4 | 11.1 | 0 |
|  | Irregular periods/vaginal bleeding | 1.3 | 22.2 | 0 |
|  | Mouth/throat sores | 1.3 | 38.1 | 0 |
|  | Nosebleed | 1.3 | 22.2 | 0 |
|  | Stretch marks | 1.3 | 22.2 | 0 |

* This item is part of the final lung cancer subset
